# Book Review: Roma Minority Youth Across Cultural Contexts: Taking a Positive Approach to Research, Policy, and Practice

**DOI:** 10.3389/fpsyg.2021.713093

**Published:** 2021-06-24

**Authors:** Guilherme Welter Wendt

**Affiliations:** Faculty of Medicine, Western Paraná State University, Francisco Beltrão, Brazil

**Keywords:** Roma minority, youth, developmental psychology, positive youth development, cross-cultural research, policy, practice

Developmental psychologists have been facing a series of paradigm shifts within the field. Not to mention the replication crisis—which sheds light to the importance of cross-cultural research encompassing non-Western, Educated, Industrialized, Rich and Democratic (WEIRD) samples–, scholars are also giving emphasis on studies about individuals' strengths. With these aspects in mind, the book “Roma Minority Youth across Cultural Contexts: Taking a Positive Approach to Research, Policy and Practice” argues that, by adopting a positive youth development (PYD) framework, our current understanding of factors associated with minority health and well-being might benefit in unique ways. Specifically, the robust collection of 13 chapters provides insight into one of the most tyrannized minority groups worldwide: the Roma minority (Dimitrova et al., 2021). This review aims to summarize the book's main contributions to the field of developmental psychology, in the light its implications for policies based on PYD not only applicable to Roma, but to other oppressed minority groups of children and adolescents.

The volume is divided into three main sections. The book begins describing the case of Roma youth nowadays in several cultural regions, both in- and outside Europe. Aside from providing abundant historical information regarding the Roma minority group, the first section also taps on policies and interventions that have been caried out with this specific population. Importantly, these reports share a common foundation: they are based on a non-deficit approach. This means that researchers did not portray their samples as “abnormal” or “deficient” when compared to “normative” youth. Indeed, it is quite clear that, even with efforts, the views and beliefs of researchers on a given topic can bias the results. Part of this is inherent to intrinsic racism, in-group influences, and an effect of the “traditional” literature from developmental textbooks. Consequently, chapters within section one report on investigations designed to promote not only better outcomes to the Roma minority itself but also to broaden the lenses of PYD among youth in social and economic vulnerability.

The second section gives the reader an in-depth view of theoretical models regarding minorities adaptation and well-being. Notably, this section contributes to efforts in defining and putting into practice the constructs related to PYD. For instance, several chapters examine how internal and external assets (i.e., positive values, social competence, social support, and community empowerment) might assist PYD-based studies. Moreover, the section highlights in what manner Roma minority groups diverge from the majority youth, which seems to be particularly timely for policy makers compromised in providing positive development of Roma and other ethnic minority groups across the globe.

In addition, the book contains a section of mixed-method studies conducted in European countries with large proportion of Roma people, including Bulgaria, Kosovo, Albania, and Serbia. Taken together, readers will notice that the samples of these studies provide a rare chance to critically reflect on the myriad of PYD implications, notwithstanding the seminal, North American roots of the PYD. However, contributors of each chapter performed comprehensive analyses of cultural-specific and further generalizable scope of their findings for those studying and working with minority groups, with examples of policy implications and suggestions of ways to guarantee the well-being for future generations of Roma minority.

Clearly, there are several interesting lessons that the book offers. By applying the PYD methodological approach with Roma youth, the work presented has implications on the knowledge about a growing, yet stigmatized and vulnerable population. Additionally, the focus on a positive approach enhances our understanding of individual (i.e., identity), cultural, and contextual (i.e., community, family) factors in Roma youth. The authors' experience with cross-cultural research provides both individual vs. comparative insights of issues regarding this minority in a variety of contexts, thus enabling inferences on experiences shared and non-shared across nations. Finally, the volume has a great potential to underpin future efforts in studying developmental processes in the face of the PYD perspective.

## Author Contributions

The author confirms being the sole contributor of this work and has approved it for publication.

## Conflict of Interest

The author declares that the research was conducted in the absence of any commercial or financial relationships that could be construed as a potential conflict of interest.
